# Transdermal tibial nerve optogenetic stimulation targeting C-fibers

**DOI:** 10.3389/fphys.2023.1224088

**Published:** 2023-06-29

**Authors:** Zhonghan Zhou, Xuesheng Wang, Xunhua Li, Limin Liao

**Affiliations:** ^1^ Shandong University, Jinan, Shandong, China; ^2^ Department of Urology, China Rehabilitation Research Center, Beijing, China; ^3^ University of Health and Rehabilitation Sciences, Qingdao, Shandong, China; ^4^ School of Rehabilitation, Capital Medical University, Beijing, China; ^5^ China Rehabilitation Science Institute, Beijing, China; ^6^ Beijing Key Laboratory of Neural Injury and Rehabilitation, Beijing, China

**Keywords:** optogenetics, tibial nerve, transdermal stimulation, bladder, rats

## Abstract

**Purpose:** To explore whether stimulation of C-fibers in tibial nerves can induce bladder inhibition by optogenetic transdermal illumination.

**Methods:** Ten rats were injected with AAV2/6-hSyn-ChR2(H134R)-EYFP into the tibial nerves. Transurethral cystometry was performed 4 weeks after the virus injection. Illumination (473-nm blue light at 100 mW) was performed with the fiber positioned above the right hind paw near the ankle. The light transmission efficiency was examined with a laser power meter. The effects on cystometry were compared before and after illumination with the bladder infused with normal saline and acetic acid, respectively.

**Result:** Upon transdermal delivery of 473-nm light at a peak power of 100 mW, the irradiance value of 0.653 mW/mm2 at the target region was detected, which is sufficient to activate opsins. The photothermal effect of 473-nm light is unremarkable. Acute inhibitory responses were not observed during stimulation regarding any of the bladder parameters; whereas, after laser illumination for 30 min, a statistically significant increase in bladder capacity with the bladder infused with normal saline (from 0.53 ± 0.04 mL to 0.72 ± 0.05 mL, *p* < 0.001) and acetic acid (from 0.25 ± 0.02 mL to 0.37 ± 0.04 mL, *p* < 0.001) was detected. A similar inhibitory response was observed with pulsed illumination at both 10Hz and 50Hz. However, illumination did not significantly influence base pressure, threshold pressure, or peak pressure.

**Conclusion:** In this preliminary study, it can be inferred that the prolonged bladder inhibition is mediated by the stimulation of C-fibers in the tibial nerves, with no frequency-dependent characteristics. Although the 473-nm blue light has limited penetration efficacy, it is sufficient to modulate bladder functions through transdermal illumination on the superficial peripheral nervous system.

## 1 Introduction

Tibial nerve stimulation (TNS), approved by the Food and Drug Administration (FDA) in 2005, is a well-established third-line therapy for overactive bladder (OAB) (Gupta et al., 2015). The precise mechanism is still unclear; however, accumulating evidence suggests that bladder inhibitory reflexes play an important role (Yecies et al., 2018; Paquette and Yoo, 2019). Evidence from animal studies indicates that the small myelinated Aδ in the afferent tibial nerve may be responsible for bladder modulation; recruiting C-fibers does not enhance the bladder-inhibitory effects further (Sato et al., 1980; Kovacevic and Yoo, 2015). This conclusion is supported by the fact that a low stimulation amplitude (4–10T_mot_) was sufficient for inhibiting OAB, the range of which was consistent with the Aβ-fiber activation threshold. However, recently a study revealed that unmyelinated C-fibers, not Aδ or Aβ, were recruited during TNS in a continuous-fill rat model. The authors argued that repeated bladder filling and emptying reinforces excitatory neural circuitry and propensity to void; the activation of C-fibers under the continuous-fill bladder condition (at 100T_mot_) is required for eliciting inhibitory responses (Paquette and Yoo, 2019). There are also studies suggesting that stimulating C fibers may lead to bladder excitation (McPherson, 1966; Kovacevic and Yoo, 2015). These inconsistent results indicate that the role of C fibers in the tibial nerve remains unclear.

Optogenetics is an emerging technology that enables the activation or inhibition of specific cells with light (Zhou and Liao, 2021). The expression of the light-sensitive protein, opsin, is genetically encoded, providing a way to target the neurons of interest. Channelrhodopsin 2 (ChR2) is the most commonly employed unselective depolarizing opsin. Exposure to blue light with a wavelength of 473 nm can induce a structural conformational inversion of 11-cis-retinal to all-trans-retinal, resulting in the opening of channels, the influx of positive ions, depolarization of the membrane, and activation of neurons. Optogenetics has revolutionized our ability to influence neural activity with high precision. Previous studies reported that AAV2/6 was largely specific to unmyelinated nociceptive neurons (Iyer et al., 2014). In this study, we selectively expressed ChR2 in C-fibers, and investigated whether stimulation of C-fibers in tibial nerves can induce bladder inhibition, bladder activation, or neither. Moreover, instead of traditional optogenetics that requires an invasive light-delivery device to be surgically implanted in the body (Zhou and Liao, 2021), we utilized transdermal illumination as a non-invasive approach to target superficial nerves, and subsequently discussed the potential of this method as an alternative or supplementary approach to traditional neural electrical stimulation.

## 2 Materials and methods

### 2.1 Experimental animals

Ten female Sprague-Dawley rats (180–220 g [g]) were used in this experiment. Animals were housed under standard conditions with food and water *ad libitum*. All procedures were performed in accordance with protocols approved by the Capital Medical University Laboratory Animal Welfare and Ethical Review Board (permit number, AEEI-2022-102).

### 2.2 Virus injection

The AAV2/6-hSyn-ChR2(H134R)-EYFP and AAV2/6-hSyn-EYFP were purchased from OBIO Technology Co. (China), which is reported to target nociceptive neurons (Iyer et al., 2014). During surgery, rats underwent anesthesia with 2% pentobarbital (30 mg/kg) by intraperitoneal injection. Breathing was monitored, and body temperature was maintained with a heating plate throughout the experiment. The tibial nerve was accessed through the medial side of the right hind leg near the ankle. The nerve was removed from the underlying fascia under a microscope. A 30-gauge Hamilton syringe (10 μL) was inserted under the epineurium of the nerve, and a total of 5×10^10^ vector genomes were injected. Fast Green (1%; 1 μL) was added to visualize the injected solution. After the injection, the incision was closed with sutures, and the rats were treated with ampicillin sodium for 3 days.

### 2.3 Cystometry

Transurethral cystometry under urethane anesthesia (1.2 g/kg, intraperitoneal) was performed 4 weeks after virus injection, as described previously (Kashyap et al., 2018). A polyethylene catheter (PE-50) was inserted transurethrally into the bladder, and the catheter was connected by a three-way stopcock to a micro-infusion pump (Stoelting, IL) and pressure transducer (MP150; Biopac, CA). The bladder was filled with normal saline (NS) at a rate of 0.08 mL/min. The bladder capacity (BC) was defined as the volume of infusion that triggered the first micturition waves and corresponding urine leakage. Bladders were emptied by gently pressing on the abdomen. The average of at least 3 BCs was measured after the stabilization of the cystometry. An OAB model was induced by infusion of 0.25% acetic acid (AA). A significant decrease in BC indicated successful disease induction.

### 2.4 Blue light illumination

A blue light was generated from a 473-nm laser with a 100-μm optical fiber (1–100 mW, RWD Life Science Co., Shenzhen, China). Illumination (100 mW, constant/10Hz/50Hz) was performed with the fiber positioned 1 cm above the right hind paw near the ankle, with the fur shaved. Acute bladder responses (during the illumination) and prolonged bladder responses (after illumination for 30 min) were both explored. An LP10 laser power meter (Sanwa Electric Instrument Co., Tokyo, Japan) was used to assess light transmission efficiency. An SDR473 radiometer (Shenzhen Speedre Technology Co., Guangzhou, China) was used to assess the light intensity. A thermocouple sensor (K-type) connected to a digital thermometer (DM6801A) was used to detect temperature changes under illumination. The tip of the thermocouple sensor was inserted into the target tibial nerve region or placed at the skin surface for local temperature detection.

### 2.5 Immunofluorescence assessment

The rats were sacrificed after cystometry, and the tibial nerves and L6-S1 dorsal root ganglions (DRGs) were obtained immediately. Tissues were fixed with 4% PFA and then embedded with OCT compound. Tissues were washed in OBS and incubated with blocking solution. Primary antibodies (1:100 Anti-Peripherin, ab246502) were diluted in blocking solution and incubated in sections. Slides were washed 3 times and incubated with secondary antibodies (1:200 Goat anti-rabbit IgG Alexa Fluor 488). Samples were imaged using a DMI8 microscope (Leica).

### 2.6 Statistics

Statistical analysis was performed using R (version 4.2.0; The R Foundation, Vienna, Austria). Results are expressed as means ± SEM. A paired *t*-test was used to compare the cystometry parameters before and after transdermal illumination. A value of *p* < 0.05 was considered statistically significant for all comparisons.

## 3 Results

### 3.1 Penetrating ratio and photothermal effect of a 473-nm light

**FIGURE 1:**
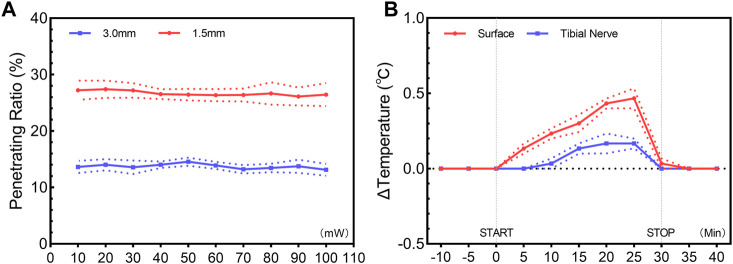
Penetration ratio **(A)** and photothermal effect **(B)** of transdermal illumination. **(A)** Penetration ratio of 473-nm constant illumination through skin and subcutaneous tissue, showing a ratio of 26.7% ± 0.2% and 13.7% ± 0.2% at a depth of 1.5 mm and 3.0 mm, with little dependence on the incident intensity. **(B)** Temporal change of temperature for skin surface and target tibial nerve region, showing the unremarkable photothermal effect of a 473-nm constant illumination. Data are presented as mean ± SEM (n = 3 for all experiments).

Previous studies have revealed the relatively limited penetration depth of visible light in biological tissues (Ash et al., 2017). Our results revealed a penetrating ratio of 26.7% ± 0.2% at a depth of 1.5 mm and 13.7% ± 0.2% at a depth of 3.0 mm, showing little dependence on the incident intensity (Figure 1A). Upon transdermal delivery of 473-nm light at a peak power of 100 mW, the irradiance value of 0.653 mW/mm^2^ at the target region was detected. Although the tissue penetration of the 473-nm light was limited, for the superficial tibial nerve at the hind leg near the ankle, the light is sufficient to activate ChR2 for cation influx (Chen et al., 2018). The photothermal effect of a 473-nm light was unremarkable. After illumination of 30 min, only a slight temperature increase at the skin surface (0.47°C ± 0.09°C) and the target region (0.16°C ± 0.04°C) were detected (Figure 1B). Based on these results, we confirmed it is possible to manipulate the tibial nerve through transdermal illumination.

### 3.2 Acute bladder responses under illumination

**FIGURE 2:**
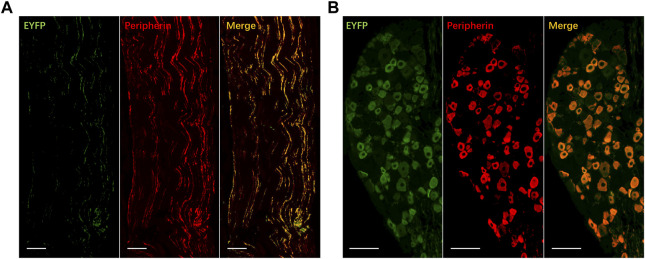
Expression of EYFP for ChR2 transfection in tibial nerve **(A)** and dorsal root ganglion **(B)**. ChR2-EYFP: green; Peripherin: red, marker for C-fibers; Co-expressed: yellow. Bar: 100 μm.

**FIGURE 3:**
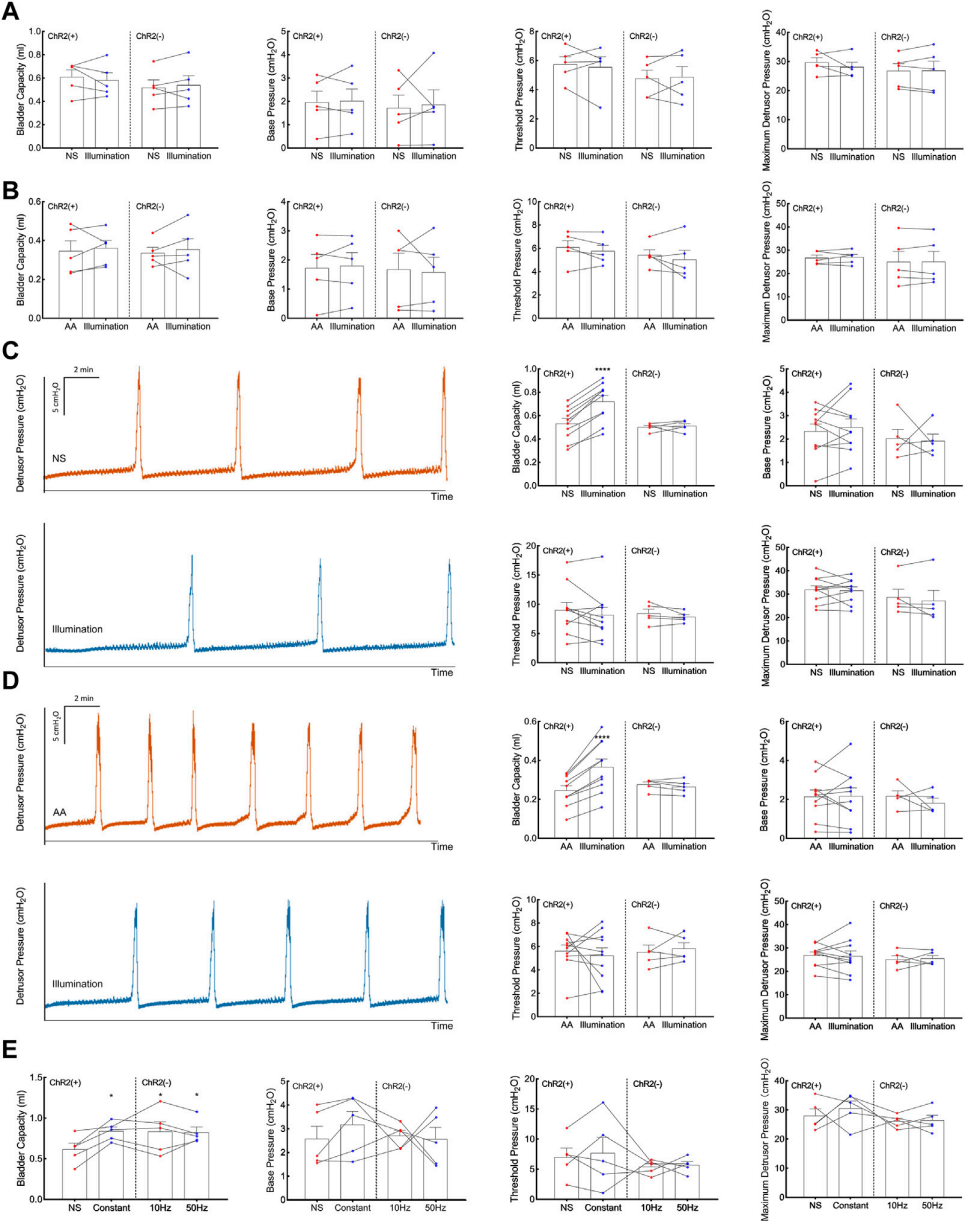
Acute and prolonged bladder responses to 473-nm illumination. **(A)** Acute bladder responses during illumination on the physiologic (normal saline-infused) bladder. **(B)** Acute bladder responses during illumination on the pathologic (acetic acid-infused) bladder. **(C)** Prolonged bladder responses after constant illumination for 30 min on the physiologic (normal saline-infused) bladder. **(D)** Prolonged bladder responses after constant illumination for 30 min on the pathologic (acetic acid-infused) bladder. **(E)** Prolonged bladder responses under different illumination protocols (Constant, pulsed 10Hz, pulsed 50Hz, 30 min). Data are presented as mean ± SEM. *: *p* < 0.05; ****: *p* < 0.0001.

Expression of ChR2-EYFP in C-fibers was confirmed by the immunohistology of the tibial nerves and DRGs 4 weeks after transfection (Figure 2). We tested whether transdermal tibial nerve optogenetic stimulation targeting C-fibers influences bladder activity. Acute inhibitory responses were not observed during infusion with NS regarding any of the bladder parameters (Figure 3A). The irritation of AA successfully induced the OAB model, with the BC from 0.61 ± 0.05 mL to 0.35 ± 0.05 mL (*p* = 0.042). Similarly, in the OAB rats, no acute bladder responses were detected during illumination (Figure 3B).

### 3.3 Prolonged bladder responses after illumination for 30 min

After illumination for 30 min in the physiological bladder, a statistically significant increase of BC (0.72 ± 0.05 mL) compared with baseline (0.53 ± 0.04 mL, *p* < 0.001; Figure 3C) was detected. The results showed in pathologic bladder a statistically significant increase of BC after illumination of the tibial nerve for 30 min (0.37 ± 0.04 mL) compared with baseline (0.25 ± 0.02 mL, *p* < 0.001, Figure 3D). Illumination did not significantly influence base pressure, threshold pressure, or peak pressure in both physiologic and pathologic conditions. We furtherly explored whether the inhibitory response was associated with illumination frequency. Continuous stimulation, pulsed 10Hz stimulation, and 50Hz stimulation all elicited an inhibitory response in BC, with no significant differences among different stimulation protocols (Figure 3E).

## 4 Discussion

### 4.1 The functional role of C-fibers in tibial nerves

The functional role of different fiber types is still not clear. Previous studies revealed that the Aδ-fibers were activated under the required stimulation intensity for TNS(4–10 T_mot_), while C-fibers were activated in a much higher range (100–200 T_mot_) (Sato et al., 1980; Kovacevic and Yoo, 2015). Based on these results, it is inferred that the inhibitory responses were mediated by Aδ fibers rather than C-fiber afferents. However, a recent study revealed that TNS leads to bladder-inhibitory effects only at stimulation amplitudes that electrically recruited unmyelinated C-fibers (Paquette and Yoo, 2019). Similarly, Wallace et al. found that stimulation of C-fibers can relieve bladder pain. What’s more intriguing is that Mapherson et al. reported bladder excitation evoked by applying ice to the hind paws in cats, which was attributed to C-fibers activated by low-temperature input (McPherson, 1966; Kovacevic and Yoo, 2015). Based on these results, it is still unknown whether stimulating C fibers are inactive, inhibitory, or excitatory in nature. In previous studies, the activation of different fiber types was achieved by choosing the electrical stimulation intensity. However, electrical stimulation lacks selectivity in nature. Optogenetics provides a powerful tool to target C-fibers in a high spatial precision manner. Previous studies have revealed that AAV2/6 has a preference for transducing unmyelinated fibers (Yu et al., 2013; Kudo et al., 2021). Our immunofluorescence result also suggests that most ChR2 expression is detected on peripherin-positive fibers and neurons, and the “off-target effect” of AAV2/6 transfection is relatively low. In this preliminary study, we found that illumination of C-fibers can both induce prolonged bladder inhibitory responses when the bladder was infused with NS and AA, respectively.

### 4.2 Absence of acute inhibitory responses

Previous experimental studies have shown that TNS can elicit acute inhibitory effects in rats (Su et al., 2012a; Su et al., 2015)and cats (Tai et al., 2011a; Tai et al., 2011b), with the mechanism still largely unknown. In contrast, our results did not show any significant acute responses in all bladder parameters. The acute inhibitory effect observed during electrical stimulation appears to differ from prolonged bladder inhibition in our study (Paquette and Yoo, 2019). The former acts like an “on-and-off” switch and can take only a few seconds or minutes to observe the influence on the bladder, whereas the latter indicates a different mechanism of neuromodulation. The illumination directly leads to the depolarization of the membrane and activation of C-fibers, while the exact mechanisms of the responses remain unclear. Previous studies revealed that intense stimulation of C-fibers may exert efferent effects, secreting neuropeptide, including substance P, calcitonin gene-related peptide, neurokinin, et al., and recruiting other fibers in the periphery (Lao and Marvizon, 2005; Adelson et al., 2009; Edvinsson et al., 2019). Further investigation is needed to explore whether the prolonged stimulation of C-fibers in the tibial nerve also affects bladder function through this mechanism. Prolonged inhibition may resemble the long-term depression of the central micturition reflex, which requires more time to develop (Jiang and Lindstrom, 1998; Su et al., 2012b). The signals may be transmitted to the spinal cord and then to higher regions in the central nervous system. Research has shown that electrical stimulation of peripheral nerves can lead to an increase in endogenous opioid peptide levels within the central nervous system (Yaksh and Elde, 1981; Wang et al., 2005). Of specific note, the level of stimulation required to produce this opioid peptide release is one that activates small nociceptive fibers. It can be inferred that the prolonged bladder inhibition may be mediated by the stimulation of C-fibers in the tibial nerves, but the recruitment and activation of other nerve fibers as well as the upstream targets remain unclear. More research is required to elucidate this potential peripheral and central mechanism in the future.

### 4.3 Consideration of stimulation frequency

The bladder reflex elicited by electrical stimulation of tibial nerves exhibits frequency-dependent characteristics, where an inhibitory response can be obtained at low frequencies (5 Hz–30 Hz in rats and cats) and an excitatory response can be evoked at high frequencies (50 Hz) (Kovacevic and Yoo, 2015). We found that both 10 Hz and 50 Hz illumination resulted in an inhibitory effect similar to constant illumination. The excitatory response was not observed at high frequencies. It appears that evoking excitatory response by TNS depends on multiple factors, and the underlying mechanism remains unknown. In this optogenetic experiment, we directly manipulate C-fibers, which excluded the possible impact of frequency and intensity on the selective activation of nerve fibers. The bladder responses elicited by the excitation of C-fibers did not exhibit frequency-dependent characteristics, indicating that there may be other mechanisms underlying bladder excitation at specific electrical frequencies.

### 4.4 Concerns of further clinical application

Optogenetics has become a powerful cell type-specific tool that enables light-mediated activation or inhibition of neural functions (Zhou and Liao, 2021). Optogenetic modulation is a potential alternative to electrical modulation. There is still a long way to go before it can be applied clinically. Traditionally, optogenetic manipulation requires surgical device implantation of tethered fiber optics or microscale light-emitting diodes, which is invasive and limited the feasibility of clinical applications. Transdermal illumination provides a noninvasive way to achieve optogenetic control of superficial peripheral afferents or muscles. It’s known that blue light with a wavelength of 473 nm has limited penetration. Our research confirmed the acceptable penetrating ratio of 473-nm blue light, which is sufficient to activate ChR2 for tibial nerve stimulation in rats. Although we confirmed the feasibility of transdermal illumination using a 473-nm blue light, there are still concerns about the limited penetration depth, especially when considering application in human beings. Many attempts have been made to improve the optogenetic activation of deep tissues. Maimon et al. (2017) revealed that higher virus concentrations can deliver more transgene copies to the neuron genome; therefore, increased density of ChR2 channels can lead to a lower required fluence rate for activation. The absorption and scattering coefficients of red light are significantly lower than those of blue light. Chrimson (Urmann et al., 2017) and ReaChR (Lin et al., 2013) were two red-shifted channelrhodopsin with a wavelength peak of 600–700 nm. These two opsin variations enable the activation of deeper tissues and can reduce the required illumination power. Near-infrared light (NIR), with a wavelength of 650–1350 nm, achieves a maximal penetration depth. Chen et al. (2018) developed transcranial NIR optogenetic stimulation of deep-brain tissues via lanthanide-doped upconversion nanoparticles (UCNPs), which can convert NIR photons into visible photons. The emission of UCNPs can be restricted to a specific wavelength using selective lanthanide-ion doping. The Yb^3+^-Tm^3+^ couple emits blue light compatible with activation of ChR2, while the Yb^3+^-Er^3+^ couple leads to green light for activation of halorhodopsin (NpHR) or archaerhodopsin (Arch). Hong (2020) developed an ultrasound-mediated deep-tissue light source for optogenetics. They made zinc sulfide nanoparticles doped with silver and cobalt, which can trap electrons and store energy until triggered by focused ultrasound and emitting blue light. Compared with direct light illumination, focused ultrasound enables a penetration depth of roughly 5 cm. These approaches provide a possible solution for noninvasive neuromodulation of the bladder, including the targets of the sacral nerve, pudendal nerve, or direct bladder smooth muscle cells (Park et al., 2017).

There are still challenges and limitations to be solved before further application of transdermal illumination of the peripheral nerves. First, the expression of ChR2 usually decreases after several weeks or months. New stable viruses should be developed to achieve persistent expression. Second, the thickness of skin and subcutaneous fat as well as the angle of the incident beam affects opsin activation. Improvement of the light-delivery device would decrease exposure variation. Third, exploring a more noninvasive and effective transducing route rather than intranerve injection is essential for translation into clinical practice. Recently, attempts have been made to introduce optogenetics as a therapeutic approach in humans, including the PIONEER study (NCT03326336) (Sahel et al., 2021). We believe such investigations can reveal the prospect of future therapeutic strategies.

## 5 Conclusion

In this preliminary study, we optogenetically stimulated C-fibers in the tibial nerves. It can be inferred that the prolonged bladder inhibition is mediated by the stimulation of C-fibers in the tibial nerves, with no frequency-dependent characteristics. Although a 473-nm blue light has limited penetration efficacy, it is sufficient to modulate bladder functions through transdermal illumination on the superficial peripheral nervous system. More effects need to be paid to this approach before we see the dawn of clinical translation for optogenetics.

## Data Availability

The raw data supporting the conclusion of this article will be made available by the authors, without undue reservation.
